# Cross-continental analysis of coastal biodiversity change

**DOI:** 10.1098/rstb.2019.0452

**Published:** 2020-11-02

**Authors:** Gavin M. Rishworth, Janine B. Adams, Matthew S. Bird, Nicola K. Carrasco, Andreas Dänhardt, Jennifer Dannheim, Daniel A. Lemley, Pierre A. Pistorius, Gregor Scheiffarth, Helmut Hillebrand

**Affiliations:** 1 Institute for Coastal and Marine Research, Department of Botany, Nelson Mandela University, Port Elizabeth 6031, South Africa; 2 Department of Zoology, Nelson Mandela University, Port Elizabeth 6031, South Africa; 3 Department of Zoology, University of Johannesburg, Auckland Park, Johannesburg 2006, South Africa; 4 School of Life Sciences, University of KwaZulu-Natal, Durban 4000, South Africa; 5 Lower Saxon Wadden Sea National Park Authority, Virchowstr. 1 26382 Wilhelmshaven, Germany; 6 Alfred Wegener Institute, Helmholtz Centre for Polar and Marine Research, Am Handelshafen 12 D-27570 Bremerhaven, Germany; 7 Helmholtz-Institute for Functional Marine Biodiversity at the University of Oldenburg [HIFMB], Ammerländer Heerstrasse 231 26129 Oldenbburg, Germany; 8 Institute for Chemistry and Biology of Marine Environments [ICBM], Carl-von-Ossietzky University Oldenburg, Schleusenstrasse 1 D-26382 Wilhelmshaven, Germany

**Keywords:** species turnover, dissimilarity, temporal trends, long-term monitoring

## Abstract

Whereas the anthropogenic impact on marine biodiversity is undebated, the quantification and prediction of this change are not trivial. Simple traditional measures of biodiversity (e.g. richness, diversity indices) do not capture the magnitude and direction of changes in species or functional composition. In this paper, we apply recently developed methods for measuring biodiversity turnover to time-series data of four broad taxonomic groups from two coastal regions: the southern North Sea (Germany) and the South African coast. Both areas share geomorphological features and ecosystem types, allowing for a critical assessment of the most informative metrics of biodiversity change across organism groups. We found little evidence for directional trends in univariate metrics of diversity for either the effective number of taxa or the amount of richness change. However, turnover in composition was high (on average nearly 30% of identities when addressing presence or absence of species) and even higher when taking the relative dominance of species into account. This turnover accumulated over time at similar rates across regions and organism groups. We conclude that biodiversity metrics responsive to turnover provide a more accurate reflection of community change relative to conventional metrics (absolute richness or relative abundance) and are spatially broadly applicable.

This article is part of the theme issue ‘Integrative research perspectives on marine conservation’.

## 1. Introduction

The current rate of global biodiversity change is both a major societal and scientific concern [1–4]. Nature's contribution to human wellbeing is closely connected to processes and properties of ecosystems that are being negatively affected by biodiversity loss. Whereas the recently recorded global extinctions do not necessarily constitute a ‘sixth mass extinction’ yet, the current rate of extinctions is concerning because it is orders of magnitude higher than during pre-human times [5]. Given the high proportion of species at risk of extinction owing to anthropogenic pressures on natural systems and a changing climate [6], further shifts in biodiversity are expected [2].

Species' ranges respond to climate change through altitudinal or latitudinal shifts over time [7–9]. Altitudinal and poleward distributional changes [7,8] induce novel range overlaps, influencing both the structure and functioning of communities. Marine range shifts on average are faster than terrestrial [8], and comparison to pre-human distributions shows clear correlations between the extent of shifts and the pace of climate change [9]. This redistribution of biodiversity is predicted to directly and indirectly affect human wellbeing and ecosystem services [10].

Consequently, biodiversity assessments have become a cornerstone of environmental monitoring programs evaluating ecosystem status and trends [11,12]. Triggered by the Convention on Biological Diversity [13], biodiversity is considered key in many present-day regulations, e.g. in the Marine Strategy Framework Directive (MSFD) of the European Union [14,15]. Consequently, biodiversity assessment also represents a core focus for both marine regions analysed here, exemplified by the trilateral monitoring concept for the Wadden Sea [12] or the National Biodiversity Assessment in South Africa [11].

However, it is surprisingly difficult to link the global changes in biodiversity to actual changes at the scale of local ecosystems, which are the focal units for monitoring, assessment and management. Extensive meta-analyses of time-series data concluded that there were no consistent trends in local species richness over time [16–18], with single locations showing increases, decreases or no trends at all. These analyses initiated an important debate on how monitoring results can be affected by details of site selection (and the inherent natural variability therein), duration, temporal resolution and study design [19–21]. In addition to these technical debates, it also became clear that the inconsistency in trend analysis arises from the simple univariate metrics used to assess biodiversity. In contrast, assessing biodiversity change depends on the spatial and temporal scales of processes and observation, with species richness being highly sensitive to scaling issues [22,23]. On the other hand, species richness and other univariate measures of diversity only reflect a minor component of biodiversity change [24–26]. Species richness captures only the net difference between local colonization and extinction. Alternative assessments do exist, such as measuring functional group (or ‘lifeform’) diversity [27], which accounts for species' traits rather than identities alone. We follow recent recommendations [17,24] to quantify the actual turnover in composition. Temporal turnover in species composition and dominance can be complete without altering the number of species [24], highlighting the problem with using univariate metrics of biodiversity. Furthermore, the observed turnover rates may be higher than predicted from random drift between species [17]. Recently, Blowes *et al*. [25] showed that marine systems are characterized by high turnover, through the analysis of a global database of time-series, BioTIME [28].

Based on these results, here we analyse which biodiversity assessment would allow current monitoring programs to detect and understand short- and long-term changes in community composition. We used data from two well-monitored coastal regions, the German part of the southern North Sea (with a focus on the Wadden Sea UNESCO World Heritage Site) and the coast of South Africa (spanning the coasts of KwaZulu-Natal, Eastern and Western Cape provinces). The two regions were chosen because they have comparable geomorphology as well as a history of anthropogenic impacts (e.g. maritime transport, eutrophication, commercial fishing). At the same time, the regions can be considered independent cases because of their geographical distance.

Considering the challenges of biodiversity monitoring using conventional metrics, here we apply and compare univariate and multivariate methods of biodiversity assessment to existing time-series data for plankton, benthic invertebrates, fish and birds. In order to enhance the interpretability of biodiversity metrics to assess ecosystem change, we ask (i) whether previously used analyses of trends of univariate metrics reveal information on temporal changes in marine biodiversity, (ii) how much compositional turnover between consecutive years exists for the different organism groups and (iii) how much turnover accumulates over time.

## 2. Methods

### (a) Study regions

The study area in the south-eastern North Sea region has an average depth of 20–40 m. The water column is generally well-mixed throughout the year owing to wave interactions and tidal currents. The seasonal sea surface temperature (SST) amplitude is up to 15°C within the year. Along the coastline stretches the world's largest intertidal soft-sediment area, the Wadden Sea, with a tidal amplitude of 1.4–4 m. Temperatures on the tidal flats are more variable, i.e. residual waters on intertidal flats can reach up to 32°C in summer contrasting with the development of ice every other winter [29]. Salinity ranges between 24 PSU in the estuarine areas and 35 PSU offshore. Sediments consist of sand and muddy-sand, while muddy substrate occurs mainly on the tidal flats and very close to the mainland shoreline.

South Africa's coastline encompasses cool-temperate, warm-temperate and subtropical biogeographic zones in the southwestern, southern and eastern coastlines [30]. The warm southerly flowing Agulhas Current flows in the east and the cold, northerly flowing Benguela Current flows in the west. Long-term monitored locations include both coastal nearshore locations as well as estuaries, which experience varying anthropogenic pressures (foremost being commercial fishing, maritime transport, freshwater abstraction and pollution or eutrophication) and natural drivers, well-described in the references listed in table 1.

**Table 1.** Datasets used in the analysis of species turnover, specifying the country (RSA = South Africa, GER = Germany), the region, the organism group looked at, the number of sites sampled, the maximum extent of years covered (TE), the number of unique station years (SY) and the total number of taxa reported (S).

Nr	country	region	organism	sites	TE	SY	S	ref
1	GER	Wadden Sea	macrozoobenthos	13	44	282	180	[31]
2	RSA	Zandvlei	phytoplankton	8	9	72	7^a^	[32]
3	RSA	St. Lucia	zooplankton	5	8	39	102	[33,34]^b^
4	RSA	St. Lucia	macrozoobenthos	5	8	40	40	[35]^c^
5	RSA	Swartkops	birds	6	18	88	103	^d^
6	RSA	Cape Recife	birds	1	16	16	55	^e^
7	RSA	East Kleinemonde Estuary	fish	1	10	10	30	[36]
8	RSA	Tsitsikamma National Park	fish	1	8	8	54	[37]
9	RSA	St. Lucia	shrimps	1	9	8	14	[38]
10	RSA	St. Lucia Wetland Park	corals	1	14	14	33	[39]
11	GER	Jade Bay	fish	1	13	13	59	[40]
12	GER	Langeoog	fish	1	15	15	58	[41]
13	GER	Elbe	fish	12	8	80	89	
14	GER	Wadden Sea	phytoplankton	4	13	46	239	
15	GER	Wadden Sea	birds	1	21	21	22	[42]
16	GER	North Sea	macrozoobenthos	4	47	173	292	[43]
17	RSA	Bird Island	fish	1	35	35	34	[44]

^a^The dataset addressed phytoplankton pigments only, i.e. did not resolve taxa.

^b^References are for data collection methods only: data available from N.K.C.

^c^Reference is for data collection method only: data available from M.S.B.

^d^http://cwac.adu.org.za/sites.php?province=Eastern%20Cape.

^e^http://cwac.adu.org.za/sites.php?province=Eastern%20Cape.

### (b) Data

Data comprised community composition assessments for major organism groups in both regions (table 1, electronic supplementary material, figure SOM 1), including phytoplankton and zooplankton (grouped collectively as ‘plankton’ hereafter), benthic macroinvertebrates, fish and coastal birds. Site descriptions for the sampling locations of these regions are expanded upon in the respective references (table 1). All datasets are derived from established monitoring programs and can be considered representative of the targeted taxonomic biodiversity regarding the extent and sampling frequency. Each dataset is characterized by internally consistent standards regarding sampling, laboratory analysis and taxonomic treatment. However, the resolution (periodicity, frequency, spatial scale and taxonomic identification level) differed between datasets, such that we only analysed within time-series, but did not address spatial differences by comparing data between time-series.

Each dataset reports on the presence and abundance of species (or in some cases, a coarser taxonomic level, but hereafter ‘species’ are referred to) in coastal marine and estuarine ecosystems. Some monitoring schemes comprised repeated samplings across tidal, diurnal or seasonal cycles. As we were interested in inter-annual change in biodiversity, we pooled the data to yearly averages across all samples. Datasets were checked for consistent naming of species and reporting, but otherwise used as reported (see also §4).

### (c) Statistics

We used the approach suggested by Hillebrand *et al*. [24] to quantify the amount of change in species composition. All analyses were performed in R [45,46]. We used the vegan package [47] to calculate taxon richness and the effective number of species (ENS) as measures of standing diversity, where ENS is the standardized species diversity measure assuming equal abundance in the community. ENS is much more robust against sampling and statistical issues than species richness [48]. Therefore, we used species richness only to calculate the net change in species richness between consecutive years (ΔS).

For turnover, we used the presence–absence-based Jaccard index to calculate the species exchange ratio based on richness (SERr), where 0 is no exchange of species identities, and 1 a complete overturn. We used Wishart's dissimilarity as a measure of abundance-based species exchange ratio (SERa), which is based on Simpson's index of dominance, a feature shared with ENS [24]. SERa also ranges between 0 and 1, with 0 indicating that species identity and relative abundance remain unchanged, but 1 indicating a complete exchange of species or a change in dominance structure.

Previous analyses of local biodiversity trends have used linear regressions over time to assess biodiversity change [16–18]. Despite the expectation of linear trends potentially being flawed [24], for comparability, we also calculated the temporal trends of annual ENS, net change in species richness (ΔS = immigrations – extinctions, which is the net difference between immigration and extinction), SERr and SERa. These linear models were calculated separately for each time-series (*n* = 66) per organism group at each site. For ΔS, SERr and SERa were compared between each year x to the following year x + 1. To be independent of any assumption regarding the form of potential temporal trends, we also assessed the overall variance in SERr or SERa to detect years exhibiting extraordinary turnover in species composition.

To analyse how biodiversity change accumulates over time, we calculated SERa and SERr for all combinations of years, i.e. between any year x and any consecutive year y, and plotted these against the temporal distance between y and x. The pairwise turnover between time points is expected to increase over time given that drift and directional trend would lead to a distance decay of similarity over time [17,49]. We tested how turnover corresponds to changes in richness and how it scales to distance in time.

## 3. Results

**Figure 1.**
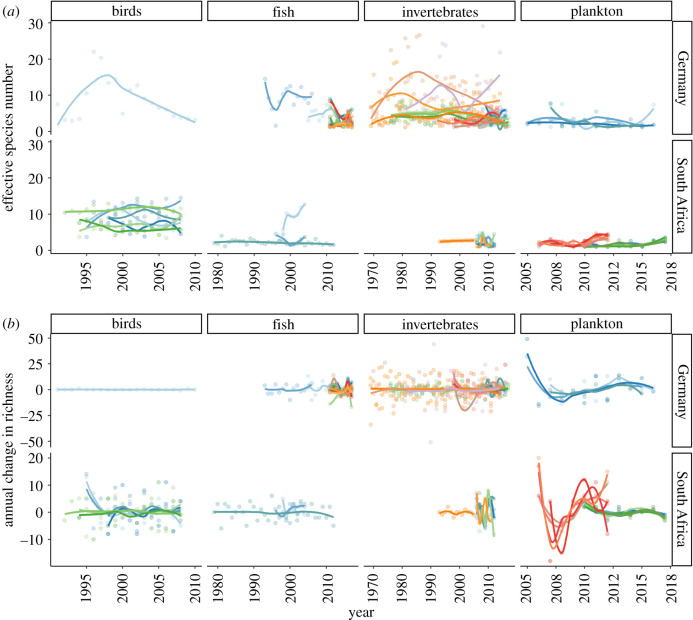
Temporal trends of the effective number of species (ENS) over time (*a*) and the annual change in richness between adjacent years (*b*), separated by organisms and regions. Each time-series is represented by differently coloured points, with loess function lines indicated to visualise the temporal dynamics. Note the change in timeframe between organisms and the different scales of the annual richness change between regions.

Effective species number (ENS) showed very high fluctuations over time (figure 1*a*), with coefficients of variation ranging from 4 to 98% of the mean. Fifteen of 66 time-series showed significant temporal trends in ENS, with 10 positive slopes and five negative slopes (electronic supplementary material, figure SOM 2B). Altogether, no consistent change of ENS over time was observed across organisms and regions.

Consequently, the annual change in richness was not different from zero (mean ± s.d. across cases: −0.7 ± 7.3) (figure 1*b*). Only two plankton time-series from South Africa showed increasingly negative ΔS over time (electronic supplementary material, figure SOM 2B).

**Figure 2.**
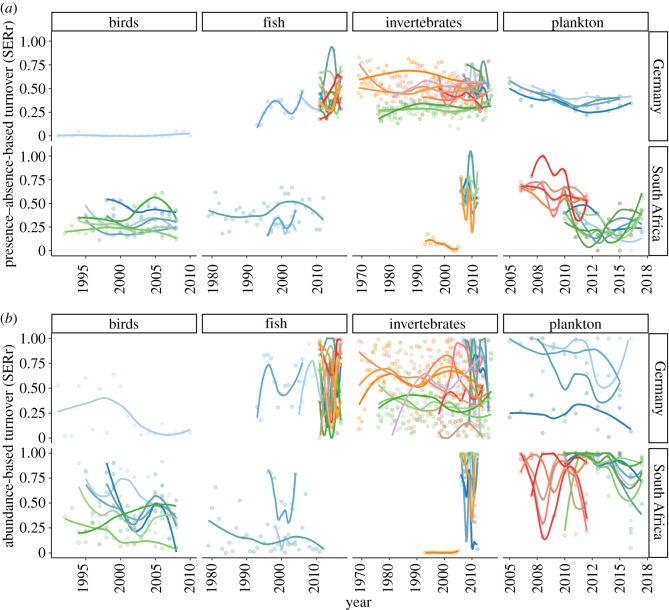
Temporal trends of the presence--absence-based turnover (SERr) (*a*) and abundance-based turnover (SERa) (*b*) between adjacent years, separated by organisms and regions. Each time-series is represented by differently coloured points, with loess function lines indicated to visualise the temporal dynamics within each sampling station.

By contrast, the multivariate assessment of species turnover showed clearer variation between sites and organisms (figure 2). Based on presence and absence of taxa, the average annual turnover was 0.29 ± 0.12 (i.e. on average, 29% of taxon identities were exchanged per year across all sites and organisms, figure 2*a*). When taking abundance into account, the yearly turnover was even larger (mean ± standard deviation = 0.52 ± 0.32), and the temporal fluctuation was higher (average CV per time-series for SERa = 48.7%, for SERr 24.5%). Given this variation, neither SERr nor SERa showed strong linear trends over time, with four time-series showing increasing turnover with time, and six showing decreases when taking presence–absence-based approaches (for SERa, two significantly positive and six significantly negative trends, respectively). Remarkably, the datasets showing consistent trends in SERr did not overlap with those showing trends using SERa.

Among organisms, invertebrates and plankton tended to have higher turnover rates than birds and fish, but this difference was small compared to the overall high variance in annual turnover (electronic supplementary material, figure SOM 3). Given this variance, the detection of extraordinary years becomes more unlikely. In fact, the only outliers for SERa all derived from the single dataset using phytoplankton pigment diversity instead of morpho-taxonomy (electronic supplementary material, figure SOM 3B). For SERr, by contrast, extraordinary years were detected (electronic supplementary material, figure SOM 3A), which indicates that such measurements can prove suitable to identify rapid changes once a time-series runs long enough to estimate the expected variance.

**Figure 3.**
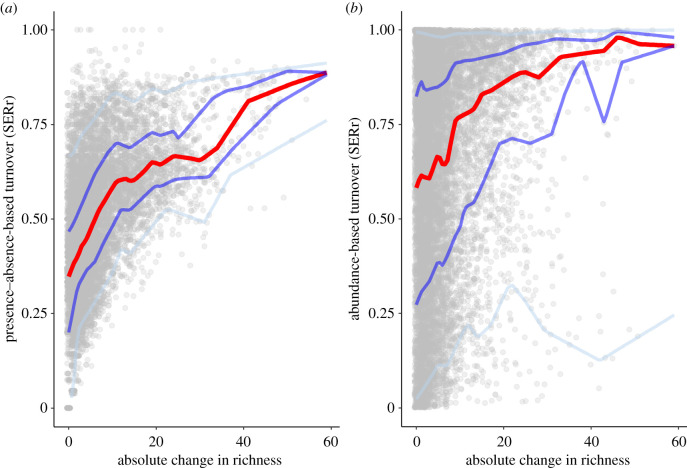
Presence--absence-based turnover (SERr) (*a*) and abundance-based turnover (SERa) (*b*) over absolute change in richness, across organism groups and regions. Symbols are all comparison of time points x to any subsequent time point in the time-series. The red lines are the medians, the dark blue lines the interquartiles (25% and 75%), the light blue lines the 5% and 95% quantiles.

Comparing all temporal data revealed that turnover can be highly independent from richness change. For presence–absence-based SERr, at zero net change in richness, any turnover between 0 and 0.65 could be observed (i.e. up to two-thirds of the community exchanged, figure 3*a*). In general, SERr and ΔS were positively related in a triangle-shaped pattern, indicating that with the increasing net change in richness, SERr becomes larger and less variable. By contrast, SERa was much more variable: the entire range of possible values of SERa between 0 and 1 were found at all levels of ΔS (figure 3*b*, see 5% and 95% quantiles). Nonetheless, median SERa also increased (and interquartile distance declined) with increasing ΔS. The fact that the full range of SERa values corresponded with no to minimal change in richness indicates that a complete reorganization of composition was possible without altering richness.

**Figure 4.**
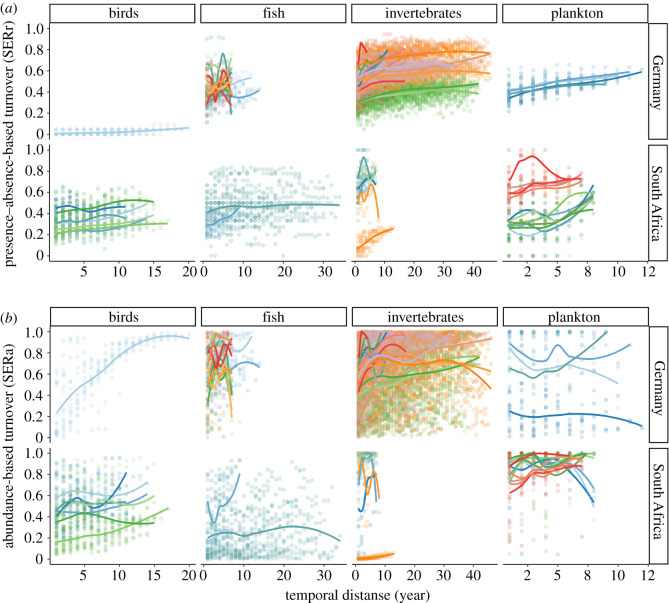
Presence–absence-based turnover (SERr) (*a*) and abundance-based turnover (SERa) (*b*) over temporal distance between years, separated by organisms and regions. Each time-series is represented by differently coloured points, with loess function lines indicated to visualise the temporal dynamics.

In most time-series, turnover accumulated, reflected by an increase in SERr or SERa, with increasing temporal distance between sampling points (figure 4). This was especially strong for SERr, indicating that replaced species assemblages did not reappear. Maximum turnover was reached after 5–15 years, which can be considered a timeframe in which community composition is fully reorganized.

## 4. Discussion

Our analyses confirmed previous reports on the scarcity of linear biodiversity trends. Significant increases or decreases in ENS, or any of the annual turnover metrics (ΔS, SERa, SERr), were present in less than 20% of time-series, thus the vast majority of the time-series showed no significant slopes. In a model attached to their recent analysis of turnover, Hillebrand *et al*. [24] stressed that linear trends are not expected in non-equilibrium conditions where immigration and extinction rates in local assemblages are independent and occur at different rates over time. Thus, any shift in environmental conditions can further increase extinction debt (fast immigration, slow extinction) or immigration credit (slow immigration, fast extinction), depending on how isolated systems are. Coastal systems additionally receive human-aided colonization of alien species [50], which on the time-scale of monitoring programmes can alter the temporal trends in diversity metrics. Thus, nonlinear dynamics are to be expected for both univariate and multivariate biodiversity metrics.

Our analysis clearly shows that the lack of linear trends in coastal biodiversity at our two study sites cannot be interpreted as a sign of no-net-change in diversity. By contrast, the average difference between consecutive years was large and showed strong variance around this mean, which indicates strong yearly fluctuations in composition (both identity and dominance). Given that we lack pre-industrial turnover data for the same regions, we cannot make statements on whether this observed turnover deviates from any pristine baseline, where turnover is only induced by inter-annual differences in abiotic conditions and random drift. Still, the observed annual turnover of 0.29 (by identity) and 0.54 (by dominance) seems large and would correspond to conditions of frequent disturbance, both short- and long-term. For the North Sea benthos, for example, most sampling sites are trawled at least once a year [51], which restricts their inhabitants to only species that can resist or tolerate this disturbance (thus no change in ENS), but induces frequent community reorganization. Annual trawling occurs randomly in this area and is constant across years [52,53] such that we cannot directly address in which state of recovery the assemblages might have been. Similarly, disturbances following alternation between drought and high-rainfall phases in the St Lucia estuarine lake system (South Africa), for example, result in regular changes in species composition [33,34,54], exacerbated by a heavy sediment load introduced from agricultural activities (sugar cane production). We intentionally did not attempt to describe the turnover metrics for each dataset as related to local conditions, focusing instead on overall trends. However, for illustration and to provide an example for the South African plankton dataset (figure 2), the fluctuations in turnover between 2006 and 2013 were as a result of a shift between communities exposed to drought (which broke in 2011) versus high-rainfall years in the St Lucia region [34].

From a monitoring perspective, rather than studying long-term changes, it may be preferable to develop methods to identify sudden changes in composition. However, the inter-annual variance in time-series is generally large, which only allows massive changes in extraordinary years to be detected. Neither the analyses of temporal trends, nor the inspection of ‘outlier years’ fulfils this quest. However, the data on SERr and SERa over temporal distance also indicate a rather gradual change in composition. Detecting such gradual changes requires consistent long-term monitoring, yielding knowledge on background variation in composition between years, and guides the evaluation of currently observed turnover. Certainly in the South African, and indeed southern hemispheric context, complete, long-term, multiple taxa monitoring datasets are rare [55], which was highlighted during our search and inclusion of datasets for this study. This is a clear and urgent management obligation that must be addressed in order to accurately quantify biodiversity variability under modern climate change scenarios. There is a trade-off between the frequency of monitoring and how much variability can be identified within an ecosystem. In rapidly changing and highly fluctuating environments, the frequency of monitoring needs to be high enough to measure the variance but the series needs to be long enough to encompass long-term shifts. The question arises whether such data—in combination with appropriate null models on random compositional drift—could foster developing a warning signal for rapid changes in marine ecosystems, which has been identified as a crucial step [56]. However, the literature on state changes in an ecosystem has mainly related to single species or environmental variables [57] and little is known on the frequency of such shifts in multispecies assemblages with a multitude of species interactions, and whether they could be detected from assemblage data.

It was beyond the scope of our analysis to link the community reorganization to certain environmental variables. However, for one factor, temperature, we recently gained better understanding of how warming increases temporal turnover and why marine communities are particularly sensitive to temperature change [58,59]. In fact, for some of the datasets, there is previous work indicating that long-term changes in composition might derive from changes in SST. This is true, e.g. for the North Sea benthos, which was affected by low SST in the 1980s, resulting in reduced abundances of warm-temperate species, which further dropped in abundance after an exceptionally cold winter in 1995/1996 [60–62]. After a biological and climate regime shift in 2000/2001, several studies observed a general increase in species number [43,63,64] with a consecutive increase in warm-temperate species and decrease in cold-temperate species at various trophic levels of the marine ecosystem and provided evidence on possible further climate-related shifts around 2010 [65]. By contrast, the faunal communities of other systems (e.g. St Lucia, South Africa) are driven by factors unrelated to temperature, such as hydrology, rainfall and sediment dynamics [33–35].

It should be noted, though, that zero turnover is neither achievable nor a relevant goal for ecosystem management, in particular when considering the importance of natural disturbances and consequent succession in community structure. Additionally, biodiversity turnover provides information only on the taxonomic restructuring of a community but provides little indication of any changes in species' traits or functional groups. Robust data on a rigorous suite of traits for most taxa are limited [66]; therefore, taxonomic data will likely remain the metric of choice for the near future in these assessments. Turnover in time is a fundamental process of community organization, and functional stability of ecosystems is often related to the fact that different species provide this function over time, i.e. turnover occurs [67]. Hodapp *et al*. [68] recently demonstrated that temporal turnover fundamentally depends on spatial heterogeneity in biodiversity: only if other species are within dispersal distance can turnover be more than a reshuffling of dominance. In coastal systems with multiple stressors, the species pool tends to shift to short-lived, fast growing and fast reproducing species on larger spatial scales [69], which induces a functional homogenization in space potentially limiting future temporal turnover.

On average, we found faster turnover in small organisms (plankton, invertebrates) than in vertebrates, which might simply reflect generation times in these organisms. Plankton biomass is closely coupled with local environmental conditions. These taxa would therefore reflect the most responsive variability or turnover following seasonal and other change. However, the difference was more dramatic for German datasets than South African data. This might imply that lower trophic levels in the German Bight are more sensitive to temperature variability whereas phyto- and zooplankton in South Africa coastal habitats (e.g. Zandvlei and St Lucia) respond rapidly to regular alternations of system states (estuarine mouth management) or hydrological conditions (associated with drought) [33,34]. Phytoplankton and invertebrate samples are more difficult to process after collection and usually reflect a higher level of local-scale patchiness than for birds or fish, for example. Our analyses did not account for this, but it is possible that the higher turnover rates (electronic supplementary material, figure SOM 3) or minimal distance–decay relationship (figure 3) might be an artefact of this rather than necessarily a feature of the communities themselves.

In our datasets, the overall setup of the monitoring was unchanged over time, such that the seasonal coverage was consistent. However, further inconsistencies in single time-series can arise from a wide range of issues during field sampling. Single samples may be lost or incomplete or weather conditions might restrict certain sampling dates or stations. Monitoring programs also often run on temporary funding regimes, which might lead to changes in numbers of subsamples taken or temporal gaps in the dataset. We did not correct for these singularities, as we were explicitly addressing how much we can learn from existing monitoring data, which requires analysing data as they are. Instead, we used approaches that are less sensitive to unknown idiosyncrasies in data collection, especially the Simpson-based metrics (ENS, SERa), which are particularly robust in this regard [48]. By using annual averages, much of the intra-annual variance will have been leveraged. Using annual averages only deals with long-term changes and does not address the question of phenology or other intra-annual shifts in biodiversity dynamics.

Our interpretations highlight that multivariate analyses of existing long-term monitoring or time-series data can be used to infer meaningful community-level patterns of change that are consistent across broad taxonomic groups and geographical scales. This represents an important baseline understanding of this relatively new proxy of biodiversity community analysis [24]. Future work would hope to expand on this by incorporating and linking these metrics of turnover with known environmental proxies and importantly comparing measures of turnover with those in pristine, comparable ecosystems or those prior to the Anthropocene using reconstructed biodiversity datasets.

## Supplementary Material

Supplementary figures

## Data accessibility

All richness and turnover data are available from the Dryad Digital Repository: https://doi.org/10.5061/dryad.vhhmgqnqp [70].
