# Supplementary material for: Cross-continental analysis of coastal biodiversity change
Source: Philos Trans R Soc Lond B Biol Sci. 2020 Nov 2;375(1814):20190452. doi: 10.1098/rstb.2019.0452 (PMC7662198; doi:10.1098/rstb.2019.0452)
Supplement: Supplementary figures [file rstb20190452supp1.docx]

Supplementary material for

Cross-continental analysis of coastal biodiversity change

Gavin M. Rishworth^1,2^, Janine B. Adams^1^, Matthew S. Bird^3^, Nicola K. Carrasco^4^, Andreas Dänhardt^5^, Jennifer Dannheim^6,7^, Daniel A. Lemley^1^, Pierre A. Pistorius^2^, Gregor Scheiffarth^8^, Helmut Hillebrand^6,7,9,*^

1. Institute for Coastal and Marine Research, Department of Botany, Nelson Mandela University, Port Elizabeth, South Africa, 6031
2. Department of Zoology, Nelson Mandela University, Port Elizabeth, South Africa, 6031
3. Department of Zoology,University of Johannesburg, Auckland Park, Johannesburg, South Africa, 2006
4. School of Life Sciences, University of KwaZulu-Natal, Durban, South Africa, 4000
5. andreas@daenhardt.com
6. Alfred Wegener Institute, Helmholtz Centre for Polar and Marine Research, Am Handelshafen 12, D-27570 Bremerhaven
7. Helmholtz-Institute for Functional Marine Biodiversity at the University of Oldenburg [HIFMB], Ammerländer Heerstrasse 231, 26129 Oldenbburg
8. Lower Saxon Wadden Sea National Park Authority, Virchowstr. 1, 26382 Wilhelmshaven, Germany
9. Institute for Chemistry and Biology of Marine Environments [ICBM], Carl-von-Ossietzky University Oldenburg, Schleusenstrasse 1, D-26382 Wilhelmshaven

**Keywords:** species turnover, dissimilarity, temporal trends, long-term monitoring

*Author for correspondence (helmut.hillebrand@uni-oldenburg.de).


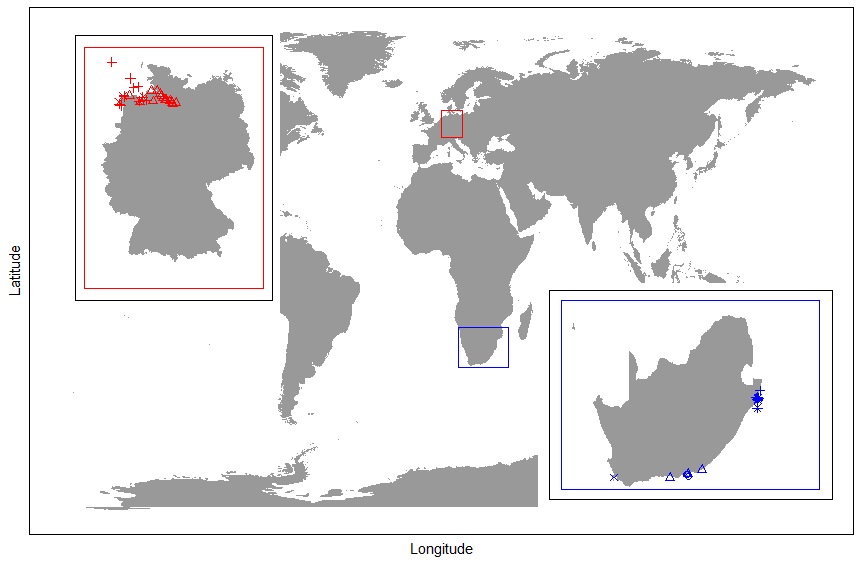


Fig. SOM1: Location of sampling sites in Germany (red) and South Africa (blue), different symbols denote fish (∆). Invertebrates (+), birds (○) and plankton (x). Please note that some sampling sites, which seemingly are on land in Germany, are from a monitoring program that explicitly included estuaries.


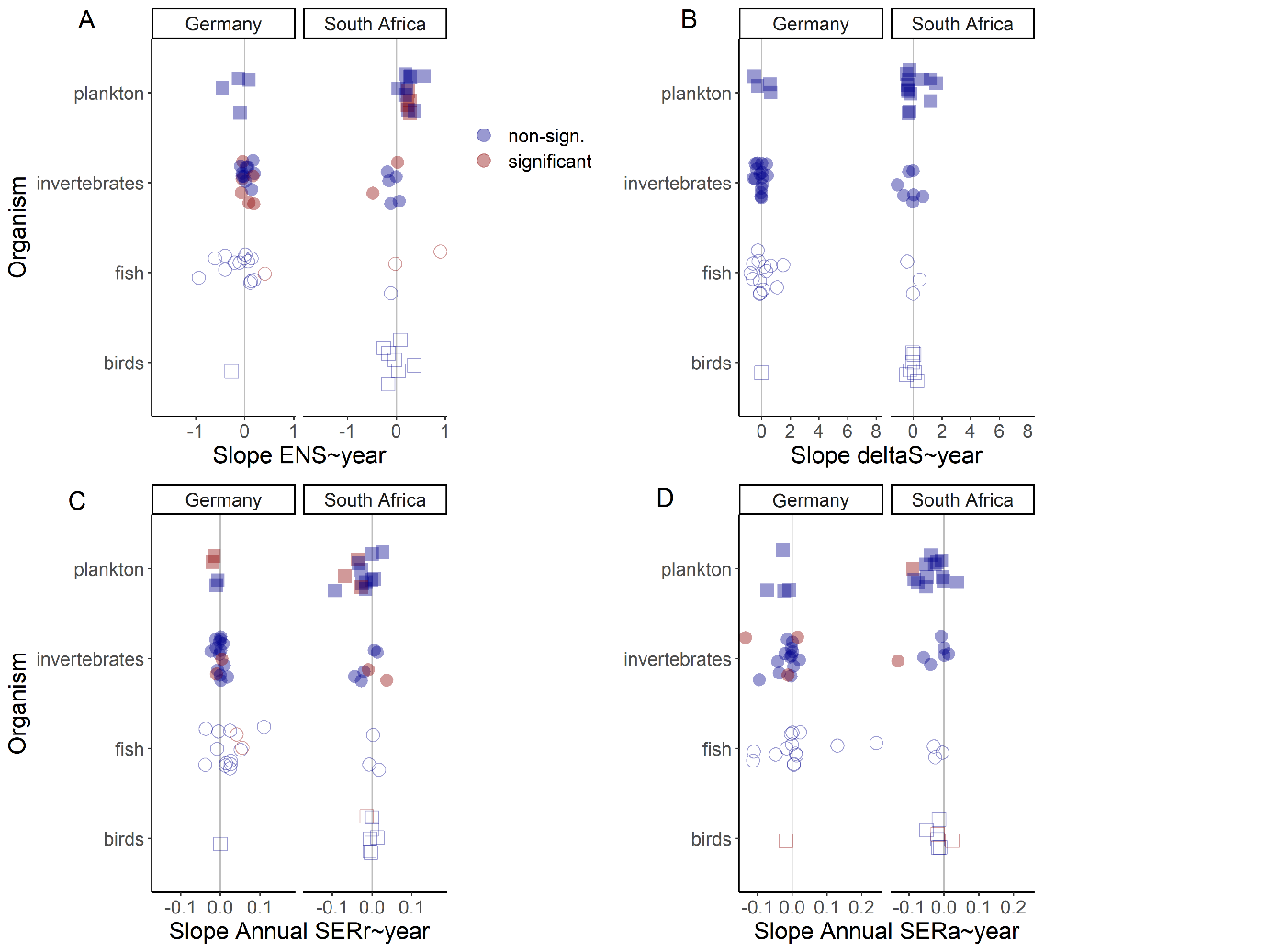


Fig. SOM 2: Temporal trends of ENS (A), annual richness change (B), presence-absence based turnover (SERr) (C) and abundance-based turnover (SERa) (D). For each data set, the slope of the linear regression is given with an indication of significance (red = significant, blue = non-significant at p = 0.05), separated by organisms and regions.


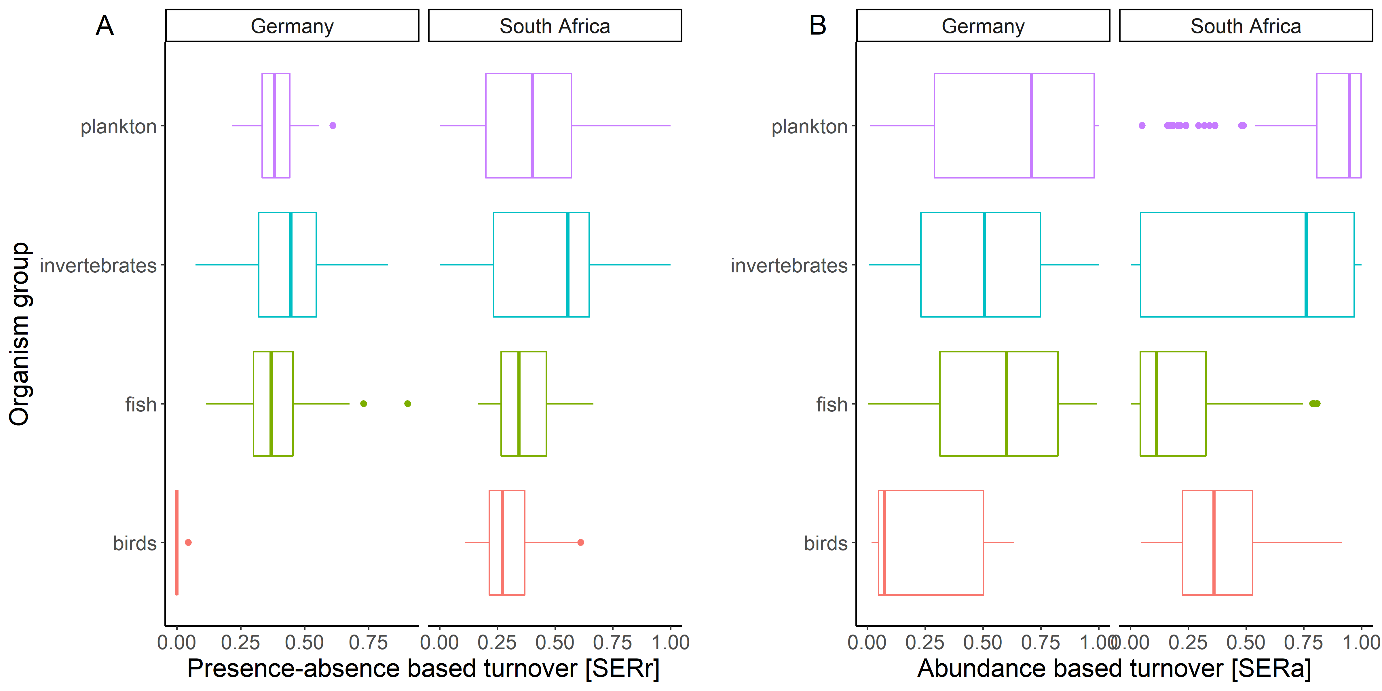


Fig. SOM 3: Boxplot of the presence-absence based turnover (SERr) (A) and abundance-based turnover (SERa) (B) between adjacent years, separated by organisms and regions. Outliers are indicated by points.
